# A multi-compartment model for interneurons in the dorsal lateral geniculate nucleus

**DOI:** 10.1186/1471-2202-12-S1-P222

**Published:** 2011-07-18

**Authors:** Geir Halnes, Sigita Augustinaite, Paul Heggelund, Gaute T Einevoll, Michele Migliore

**Affiliations:** 1 IMT, Norwegian University of Life Sciences, Ås, NO-1432, Norway; 2 Department of Physiology, University of Oslo, Oslo, NO-0317, Norway; 3 Institute of Biophysics, National Research Council, Palermo, IT-90146, Italy

## 

GABAergic interneurons (INs) in the dorsal lateral geniculate nucleus (dLGN) shape the information flow from retina to cortex, presumably by controlling the number of visually evoked spikes in thalamocortical (TC) cells, and refining their receptive field. The dLGN INs exhibit a rich variety of firing patterns. Depolarizing current injections to the soma may induce tonic firing, periodic bursting or an initial burst followed by tonic spiking, sometimes with prominent spike time adaptation. When released from hyperpolarization, some dLGN INs elicit rebound bursts, while others return more passively to the resting potential [1-3].

A full mechanistic understanding that explains the function of the dLGN on the basis of neuronal morphology, physiology and circuitry is currently lacking. One way to address this question is to develop and investigate mathematical models of the involved cells and their interactions. While detailed models of the well studied TC cells exist, previous models of the less studied dLGN INs are simplified and use only passive dendritic properties. Several ion channels are present in the dendrites of dLGN INs [4-6], and may be of particular importance in these neurons, as their dendrites have not only postsynaptic contacts for excitatory retinal input, but also presynaptic terminals for inhibitory output to TC-cell dendrites [7].

We here present a detailed compartmental model of a dLGN IN with a detailed morphology and active dendritic, as well as somatic, conductances. The simulation tool *NEURON* (http://neuron.duke.edu/) was used for the model. The conductance values were constrained by somatic voltage recordings from two dLGN INs under 8 experimental conditions. The model reproduces qualitative features in the experimentally recorded response patterns, as well as quantitative data on the firing frequency as a function of injected current. We show that relative differences in conductance values, rather than differences in ion channel composition, explain the differences in the response properties of the two neurons, and can account for the various response patterns listed above. The model lays the foundation for studying the function of the dLGN IN and its interactions with TC cells within a network context, and does also in its own right give new insights in the properties of dLGN IN activity.
